# Supplementary material for: Genome-Wide Identification and Characterization of G2-Like Transcription Factor Genes in Moso Bamboo (Phyllostachys edulis)
Source: Molecules. 2022 Aug 26;27(17):5491. doi: 10.3390/molecules27175491 (PMC9457811; doi:10.3390/molecules27175491)
Supplement: Supplementary file 1 [file molecules-27-05491-s001.zip › Table S1.pdf]

Table S1. Detailed information about *ZmGLK* genes in maize.

Gene name	Sequences ID	MW(Da)	PI	CDS Length(bp)	Size(aa)
ZmGLK1	GRMZM2G009060_T01	39861.23	7.28	1080	359
ZmGLK2	GRMZM2G006477_T01	49645.57	6.16	1353	450
ZmGLK3	GRMZM2G113742_T01	29156.18	9.43	804	267
ZmGLK4	GRMZM2G056400_T01	47557.66	8.82	1377	458
ZmGLK5	GRMZM5G887276_T03	46203.21	8.73	1305	434
ZmGLK6	GRMZM2G039074_T02	45382.56	9.8	1284	427
ZmGLK7	GRMZM2G034563_T01	26111.26	6.92	747	248
ZmGLK8	GRMZM2G374986_T01	42557.7	9.3	1176	391
ZmGLK9	AC234520.1_FGT003	19591.43	9.56	561	186
ZmGLK10	GRMZM2G124540_T01	35804.38	6.12	1017	338
ZmGLK11	GRMZM2G379656_T01	16687.42	5.15	438	145
ZmGLK12	GRMZM2G117193_T01	53169.28	7.22	1488	495
ZmGLK13	GRMZM2G125704_T02	30274.45	9.19	816	271
ZmGLK14	GRMZM2G035370_T01	51625.62	7.8	1404	467
ZmGLK15	GRMZM2G069525_T01	33135.25	9.08	939	312
ZmGLK16	GRMZM2G173882_T01	36735.53	7.02	1038	345
ZmGLK17	GRMZM5G846506_T01	41247.17	9.47	1146	381
ZmGLK18	GRMZM2G370425_T01	43773.75	6.65	1230	409
ZmGLK19	GRMZM2G348238_T01	52172	5.8	1500	499
ZmGLK20	GRMZM2G074908_T01	32180.44	5.68	915	304
ZmGLK21	GRMZM2G087804_T03	49254.78	6.72	1386	461
ZmGLK22	GRMZM2G396825_T01	32555.42	8.54	894	297
ZmGLK23	GRMZM2G175827_T02	49074.33	9.16	1407	468
ZmGLK24	GRMZM2G070865_T01	39524.16	6.71	1119	372
ZmGLK25	GRMZM2G315506_T01	37640.56	8.66	1074	357
ZmGLK26	GRMZM2G168002_T04	28660.53	6.59	813	270
ZmGLK27	AC233960.1_FGT003	30635.8	8.4	840	279
ZmGLK28	GRMZM2G052544_T01	40783.49	7.22	1131	376
ZmGLK29	GRMZM2G083472_T01	33179.87	5.69	921	306
ZmGLK30	GRMZM2G010920_T01	48305.91	6.61	1317	438
ZmGLK31	GRMZM2G477238_T01	58738.69	6.21	1623	540
ZmGLK32	GRMZM2G060834_T01	40092.95	7.41	1143	380
ZmGLK33	GRMZM2G106185_T01	40665.18	9.85	1137	378
ZmGLK34	GRMZM2G060485_T01	58845.41	6.36	1638	545
ZmGLK35	GRMZM2G379167_T01	44260.81	6.57	1209	402
ZmGLK36	GRMZM2G171468_T01	38386.96	5.33	1089	362
ZmGLK37	GRMZM2G701218_T01	46434.86	6.53	1245	414
ZmGLK38	AC219020.4_FGT002	59226.91	6.17	1665	554
ZmGLK39	GRMZM2G117854_T03	28416.91	6.08	774	257
ZmGLK40	GRMZM2G100709_T01	28085.27	7.71	771	256
ZmGLK41	GRMZM2G398055_T01	31420.01	7.09	939	312

ZmGLK42	AC155434.2_FGT005	39792.34	7.78	1104	367
ZmGLK43	GRMZM2G064197_T01	27732.51	8.48	771	256
ZmGLK44	GRMZM2G162409_T03	48417.02	5.14	1326	441
ZmGLK45	GRMZM2G173943_T02	34026.39	5.95	939	312
ZmGLK46	GRMZM2G082264_T01	48352.37	6.98	1434	477
ZmGLK47	GRMZM2G100176_T01	39865.83	7.06	1110	369
ZmGLK48	AC234155.1_FGT002	44110.34	6.23	1182	393
ZmGLK49	GRMZM2G067702_T01	23717.77	6.55	660	219
ZmGLK50	GRMZM2G471600_T01	30828.47	6.83	900	299
ZmGLK51	GRMZM2G081671_T01	37367.17	7.7	1032	343
ZmGLK52	GRMZM2G333083_T01	56361.35	6.48	1566	521
ZmGLK53	GRMZM2G454449_T01	28049.28	8.59	762	253
ZmGLK54	GRMZM2G026833_T01	50523.81	5.2	1431	476
ZmGLK55	GRMZM2G124495_T01	49168.55	8.15	1377	458
ZmGLK56	GRMZM2G016370_T01	48702.17	6.08	1362	453
ZmGLK57	GRMZM2G159119_T01	49338.97	6.58	1380	459
ZmGLK58	GRMZM2G090230_T01	35491.89	8.74	987	328
ZmGLK59	GRMZM2G123308_T02	37355.24	7.35	1071	356
