# Supplementary material for: Genome-Wide Identification and Characterization of G2-Like Transcription Factor Genes in Moso Bamboo (Phyllostachys edulis)
Source: Molecules. 2022 Aug 26;27(17):5491. doi: 10.3390/molecules27175491 (PMC9457811; doi:10.3390/molecules27175491)
Supplement: Supplementary file 1 [file molecules-27-05491-s001.zip › Table S2.pdf]

Table S2. The MEME motif sequences and lengths of PeGLK proteins in moso bamboo.

Motif	Width	Best possible match
1	29	KATPKSVLELMGVKGLTJYHVKSHLQKYR
2	29	LRTSKKPRLRWTPELHERFVDAVEQLGGP
3	33	LHEQLEVQRHLQLRIEAQGKYLQSILEKAQKAL
4	21	DSKKSMQJSEALRMQMEVQRR
5	50	VISDVHMPDMDGFKLLELVGLEMDLPVIMLSANGETKTVMKGITHGACDY
6	50	DQFPVGMRVLAVDDDPTCLKVLETLLLRCQYHVTTTGQAATALKMLRENK
7	50	HLYGGGHLELQQQMERPMDSFLAFNESCIGSVGKKSPSHYSAAGKSPMVW
8	22	LLKPVRIEELRNIWQHVVRRKF
