# Supplementary material for: Genome-Wide Identification and Characterization of G2-Like Transcription Factor Genes in Moso Bamboo (Phyllostachys edulis)
Source: Molecules. 2022 Aug 26;27(17):5491. doi: 10.3390/molecules27175491 (PMC9457811; doi:10.3390/molecules27175491)
Supplement: Supplementary file 1 [file molecules-27-05491-s001.zip › Table S3.pdf]

Table S3. The microarray data of 36 *PeGLK* genes in moso bamboo.

Name	leaf	stem	rhizome	root
PeGLK1	0.984253	0.57124	0.67832	0.53432
PeGLK4	1.12454	0.74621	0.85234	0.91527
PeGLK6	0.73225	0.83145	0.73621	1.1355
PeGLK7	1.18561	0.93672	0.83731	1.6381
PeGLK12	0.93526	0.62367	0.41963	0.67124
PeGLK20	0.962358	0.78342	0.63712	0.88523
PeGLK21	0.88724	0.61905	0.67522	1.07946
PeGLK28	0.91794	0.28798	0.21074	0.87593
PeGLK35	1.21654	0.41956	0.63621	0.65722
PeGLK36	0.96211	0.37986	0.46787	1.14839
PeGLK37	1.16443	0.38973	0.58931	0.94166
PeGLK39	0.97934	0.37014	0.25546	0.73654
PeGLK40	0.98615	0.42167	0.37024	0.55748
PeGLK44	1.11754	0.62673	0.97601	0.6172
PeGLK47	1.06945	0.91634	0.54242	0.47154
PeGLK48	1.01527	0.56953	0.3704	0.64153
PeGLK50	1.08492	0.75732	0.54156	0.57921
PeGLK51	1.0591	0.80332	0.22035	0.52156
PeGLK52	0.57052	0.8457	0.92254	0.46047
PeGLK53	0.76235	0.27563	0.84032	0.84356
PeGLK58	0.88976	0.64111	0.56832	0.42035
PeGLK59	0.91163	0.64843	0.73928	0.56857
PeGLK60	0.97385	1	0.87877	0.62037
PeGLK61	0.46549	0.33167	0.34575	0.94832
PeGLK62	0.92798	0.2285	0.55362	0.56037
PeGLK63	0.91659	1.31758	0.86778	0.85894
PeGLK65	1.03194	0.86231	0.34562	0.48031
PeGLK66	1.09692	0.81865	0.27653	0.42925
PeGLK67	1.03564	0.66952	0.84421	0.72045
PeGLK68	0.74256	0.559654	1.10452	0.73916
PeGLK69	1.05646	0.96328	0.77948	0.62739
PeGLK70	0.88795	0.35285	0.66359	0.39045
PeGLK71	0.56842	0.98253	0.70531	0.46321
PeGLK72	0.82116	0.34278	0.1782	0.60144
PeGLK75	1.11021	0.27324	0.34677	0.48055
PeGLK78	0.74623	0.38345	0.15824	0.98532
