# Supplementary material for: Genome-Wide Identification and Characterization of G2-Like Transcription Factor Genes in Moso Bamboo (Phyllostachys edulis)
Source: Molecules. 2022 Aug 26;27(17):5491. doi: 10.3390/molecules27175491 (PMC9457811; doi:10.3390/molecules27175491)
Supplement: Supplementary file 1 [file molecules-27-05491-s001.zip › Table S4.pdf]

Table S4. The primers of qRT-PCR of 37 *PeGLKs* and *PeTIP41*.

**Genes**		**Primers used in qRT-PCR(5′→3′)**
PeGLK1	Forwards	CACCAACGACGAGGTCAAGA
	Reverse	ACCTGCTCGACTGTTTCTGGA
PeGLK6	Forwards	ACGATTGCCGAAAGGCAGTA
	Reverse	GGGAACCTCTTCAACTCGCA
PeGLK7	Forwards	AGTCGTATGAGCGTGCCAAA
	Reverse	GAGCCTGTACACAGGGGAAC
PeGLK12	Forwards	GGCCCCAACCATGGACATTA
	Reverse	CCACCAAGGCCCTCGTAAAA
PeGLK20	Forwards	CGGAGACTCTGAGGACAAGC
	Reverse	CTGGGTTGCTGTGAAATGGC
PeGLK21	Forwards	AAGGCCACTACGTTGGTGAG
	Reverse	TTTCACGAGGGTCGAAAGGG
PeGLK28	Forwards	TGCACCCTTGATCCCAAGTC
	Reverse	TCCAAAGCACCAACGAGGTT
PeGLK35	Forwards	GTCGCACTTAACCCAGGGAA
	Reverse	CGCGCTTGCAAACTAGATCC
PeGLK36	Forwards	ATAAAAGCGCCGGTGATGGT
	Reverse	TCCTGGTGCATATAGCTGCG
PeGLK37	Forwards	ACGATCGAGAACTTCACCCG
	Reverse	AACGGCTCCAACCATAAGGG
PeGLK39	Forwards	GACTGTGCTGCTGACCTGAT
	Reverse	TGCTGGACATCACCCCTTTC
PeGLK40	Forwards	ACCAGTGATGCTAAGGCCAC
	Reverse	TCAAGAGGGCGTTTCAAGCA
PeGLK44	Forwards	TTGTTGAGGAGCGGGTTCAA
	Reverse	TGGGACTCTAGGCCTCACAA
PeGLK47	Forwards	ACGAGCAGCAGACATCAACA
	Reverse	AGCTCGGCACTAGCAAAGTT
PeGLK48	Forwards	GTTCACGTGGACGATCGAGA
	Reverse	AACGGCTCCAACCATAAGGG
PeGLK50	Forwards	GTCAAGGGCTGCTCAGATGT
	Reverse	CGGGAACTCGTAACGGTCAT
PeGLK51	Forwards	AGATCCAGTTTGGGTCGCTG
	Reverse	CGGCCCACCATTTGTTACAC
PeGLK52	Forwards	CCATGCGAAATCTGCAGCAA
	Reverse	TCGCGGTAACGTAAGCTCTC
PeGLK53	Forwards	AAAAGTGCCGGAGATGGTGA

	Reverse	TGGTGCATATAGCTGCGGAA
PeGLK58	Forwards	GGACTGCCTGGCACTGTATT
	Reverse	TGCCAAGCTGTTCAAATCGC
PeGLK59	Forwards	AAGAAGAGCAAGTGGTCGCA
	Reverse	AGTGTAGAGGTGCAACTGGC
PeGLK60	Forwards	TCTGCCAGGGCCAATACAAG
	Reverse	TGTAAGGAGATGGCCCGGTA
PeGLK61	Forwards	GTCGTGAGCTGCAGCAAAAA
	Reverse	TCCCTACACTCTTCGCGGTA
PeGLK62	Forwards	GAGGATAGAGTTCCACGCCG
	Reverse	CTAAGCTCCCGATCGAACCC
PeGLK63	Forwards	TGCTGCAGAGTGCACCAATA
	Reverse	TTGCTCGAGTCCATTGTGCT
PeGLK65	Forwards	CCAAGCGGCAAAACCATACC
	Reverse	CAGCGGAAGAGGTTCAAGGT
PeGLK66	Forwards	AAGAAGTGTTCCGGGTGCAA
	Reverse	CCATGGGCAGGCACTAATGA
PeGLK67	Forwards	GTTCTAAGGCCAGCGAGGTT
	Reverse	CACACTCGCAGCAAGTCAAC
PeGLK68	Forwards	CAGGGACAAAGCCGATTCCT
	Reverse	CCAGTCCCCTCCACCAGATA
PeGLK69	Forwards	CCCGATGCAGACAGGAATGT
	Reverse	TGAGGTAGCTCCTCCTCACC
PeGLK70	Forwards	GACGAGATCTACTCTGCCGC
	Reverse	GGCCTTGGGCATGTGATTTG
PeGLK71	Forwards	GCAAGGAGGAGGTTCGTTGA
	Reverse	GCTTTCGCTGCTATCTGTGC
PeGLK72	Forwards	TGCAGACTCGGTTAGCACAG
	Reverse	TGGATTGGAAGGCGAGAACC
PeGLK75	Forwards	ACTGAAGCGCGAAAGCTAGT
	Reverse	TCGCTGGTGGTGATGAAACA
PeGLK78	Forwards	ACGTCAAGTGAATGGTCGCT
	Reverse	TGGATTGGAAGGCGAGAACC
PeTIP41	Forwards	AAAATCATTGTAGGCCATTGTCG
	Reverse	ACTAAATTAAGCCAGCGGGAGTG
