# Supplementary material for: Genome-Wide Identification and Characterization of G2-Like Transcription Factor Genes in Moso Bamboo (Phyllostachys edulis)
Source: Molecules. 2022 Aug 26;27(17):5491. doi: 10.3390/molecules27175491 (PMC9457811; doi:10.3390/molecules27175491)
Supplement: Supplementary file 1 [file molecules-27-05491-s001.zip › Table S5.pdf]

Table S5.

(A) Expression level of *PeGLK* genes in response to cold stress.

Time / Gene ID	0h	1h	3h	6h	12h	24h
PeGLK1	1	2.4	4.2	2.5	4.5	3.9
PeGLK7	1.73	2.89	5.88	5.37	20.83	26.8
PeGLK21	1.32	1.56	2.81	3.95	5.02	5.11
PeGLK36	2.6	2.8	18.4	4.7	14.8	28.2
PeGLK40	6.7	12.8	20.45	35.6	43.45	38.9
PeGLK48	1	3.89	4.26	4.31	3.29	3.11
PeGLK50	1	0.58	0.82	1.31	3.18	2.55
PeGLK53	1	1	5.21	8.23	10.6	12.2
PeGLK60	1.35	1.89	22.8	40.9	80.9	35.42
PeGLK61	1	0.82	0.95	4.15	2.59	2.02
PeGLK67	1	2.61	2.02	1.54	1.86	1.72
PeGLK70	1	0.38	0.65	0.57	0.41	0.32
PeGLK72	1	2.1	3.5	7.9	15.1	22.6

(B) Expression level of *PeGLK* genes in response to osmotic stress.

Time / Gene ID	0h	1h	2h	3h	6h	12h
PeGLK1	1	0.62	0.68	3.79	2.55	2.83
PeGLK7	1	1	13.58	8.21	10.32	16.11
PeGLK21	1	1	3.71	3.55	5.09	5.32
PeGLK36	1	4.68	10.62	20.78	28.67	32.92
PeGLK40	1	1	1.84	1.38	4.71	1.59
PeGLK48	1	2.8	1.6	2.34	3.39	2.59
PeGLK50	1	0.82	1.73	2.31	2.02	1.58
PeGLK53	1	1.77	4.32	7.11	9.29	3.51
PeGLK60	1	0.53	0.99	0.82	4.85	2.58
PeGLK61	1	0.62	0.55	0.58	0.42	0.47
PeGLK67	1	1.94	8.32	6.11	12.78	22.72
PeGLK70	1	18.91	20.78	25.8	52.91	10.56
PeGLK72	1	0.84	0.63	0.59	0.52	0.38

(C) Expression level of *PeGLK* genes in response to MeJA treatment.

Time / Gene ID	0h	1h	3h	6h	12h
PeGLK1	1	2.4	4.2	6.5	14.5
PeGLK7	1	22.34	35.67	60.67	56.45
PeGLK21	1	0.56	0.78	0.85	0.62
PeGLK36	1	2.1	3.5	7.9	15.1
PeGLK40	1	7.83	10.54	15.2	12.45
PeGLK48	1	3.61	4.02	3.54	3.86

PeGLK50	1	5.73	6.32	13.45	10.79
PeGLK53	1	3.89	5.88	7.37	15.83
PeGLK60	1	3.41	4.55	4.27	3.22
PeGLK61	1	5.23	8.42	16.31	12.66
PeGLK67	1	2.46	3.42	4.15	4.67
PeGLK70	1	5.38	6.84	10.78	12.52
PeGLK72	1	3.42	3.55	4.33	5.33

(D) Expression level of *PeGLK* genes in response to GA treatment.

**Time / Gene ID**	**0h**	**1h**	**3h**	**6h**	**12h**
PeGLK1	1	2.12	2.98	3.55	3.18
PeGLK7	1	2.89	1.56	3.37	4.83
PeGLK21	1	2.56	3.86	4.59	4.57
PeGLK36	1	0.79	0.83	1.46	1.83
PeGLK40	1	4.67	4.22	3.69	3.22
PeGLK48	1	0.85	2.55	3.41	4.22
PeGLK50	1	3.54	4.66	7.62	7.81
PeGLK53	1	10.89	18.33	26.51	38.31
PeGLK60	1	2.16	3.42	3.89	3.36
PeGLK61	1	0.95	2.59	3.15	3.59
PeGLK67	1	0.74	0.85	0.53	0.62
PeGLK70	1	0.67	0.89	0.73	0.79
PeGLK72	1	0.38	0.65	0.57	0.41
