# Supplementary figures and images for: Genome-Wide Identification and Characterization of G2-Like Transcription Factor Genes in Moso Bamboo (Phyllostachys edulis)
Source: Molecules. 2022 Aug 26;27(17):5491. doi: 10.3390/molecules27175491 (PMC9457811; doi:10.3390/molecules27175491)

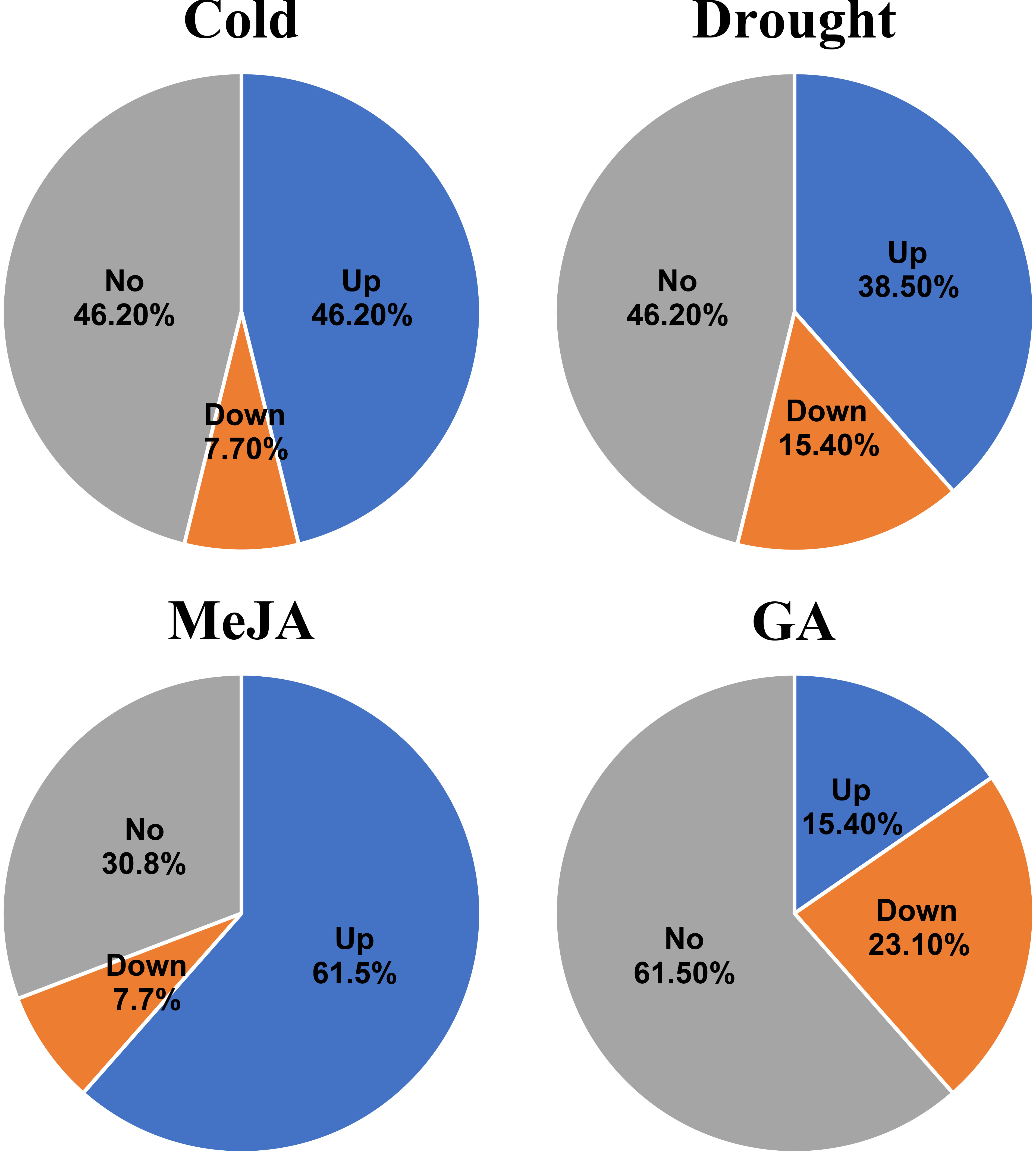

Supplement: Supplementary file 1 [file molecules-27-05491-s001.zip › Figure S2Percentage of PeGLKs response to abiotic stress and hormone treatments. Up and Down represent up and down regulated after cold stress, o.jpg]
